# MRI detection of free‐contrast agent nanoparticles

**DOI:** 10.1002/mrm.30292

**Published:** 2024-09-29

**Authors:** Francesca Garello, Eleonora Cavallari, Martina Capozza, Marta Ribodino, Roberta Parolisi, Annalisa Buffo, Enzo Terreno

**Affiliations:** ^1^ Molecular and Preclinical Imaging Centers, Department of Molecular Biotechnology and Health Sciences University of Turin Turin Italy; ^2^ Department of Neuroscience “Rita Levi Montalcini” University of Turin Turin Italy; ^3^ Neuroscience Institute Cavalieri Ottolenghi University of Turin Orbassano Italy

**Keywords:** CSI, liposomes, micelles, MRI, nanoemulsions, nanoparticles

## Abstract

**Purpose:**

The integration of nanotechnology into biomedical imaging has significantly advanced diagnostic and theranostic capabilities. However, nanoparticle detection in imaging relies on functionalization with appropriate probes. In this work, a new approach to visualize free‐label nanoparticles using MRI and MRS techniques is described, consisting of detecting by ^1^H CSI specific proton signals belonging to the components naturally present in most of the nanosystems used in preclinical and clinical research.

**Methods:**

Three different nanosystems, namely lipid‐based micelles, liposomes, and perfluorocarbon‐based nanoemulsions, were synthesized, characterized by high resolution NMR and then visualized by ^1^H CSI at 300 MHz. Subsequently the best ^1^H CSI performing system was administered to murine models of cancer to evaluate the possibility of tracking the nanosystem by looking at its proton associated signal. Furthermore, an in vitro comparison between ^1^H CSI and ^19^F MRI was carried out.

**Results:**

The study successfully demonstrates the feasibility of detecting nanoparticles using MRI/MRS without probe functionalization, employing ^1^H CSI. Among the nanosystems tested, the perfluorocarbon‐based nanoemulsion exhibited the highest SNR. Consequently, it was evaluated in vivo, where its detection was achievable within tumors and inflamed regions via ^1^H CSI, and in lymph nodes via PRESS.

**Conclusions:**

These findings present a promising avenue for nanoparticle imaging in biomedical applications, offering potential enhancements to diagnostic and theranostic procedures. This non‐invasive approach has the capacity to advance imaging techniques and expand the scope of nanoparticle‐based biomedical research. Further exploration is necessary to fully explore the implications and applications of this method.

## 1 INTRODUCTION

Nanotechnology has certainly revolutionized many human activities. In life sciences, the design of nanometric materials has allowed the achievement of important milestones, including the development of new and more effective diagnostic and therapeutic strategies, as very recently demonstrated by how scientists have successfully addressed the urgent necessity to promptly produce a coronavirus disease 2019 (COVID‐19) vaccine on a worldwide scale.^1^, ^2^, ^3^

Of course, also biomedical imaging greatly benefited from these advances. The use of nanoparticles has positively impacted the research in this field over the past decades because of the many advantages that nanosystems may confer to imaging probes in terms of enhancing the sensitivity in contrast detection, improving pharmacokinetics, and facilitating multimodal imaging capability, thus significantly boosting the overall potential of such agents to design more and more advanced and sophisticated diagnostic/theranostic procedures.^4^, ^5^

However, the detection of a nanoparticle by any of the currently available in vivo imaging technologies inevitably requires the inclusion in the nanosystem of a proper chemical entity capable of generating a specific signal to be localized by an external detector. Typical examples are represented by (super)paramagnetic,^6^, ^7^ fluorine‐containing,^8^ or hyperpolarized probes for MRI/magnetic particle imaging (MRI/MPI) detection,^9^ chromophores/fluorophores for photoacoustic/fluorescence imaging,^10^ and radioisotopes for PET/single photon emission computed tomography (PET/SPECT) detection.^11^ Though the incorporation of an imaging unit in a nanosystem is not generally a difficult task, in some circumstances, especially when the probe is conjugated on the outer surface of the particle, it may affect the pharmacokinetic properties of the system. In addition, for regulatory purposes, the introduction of a chemical entity in a nanocarrier, even when both partners are approved for clinical use, requires the submission of a new authorization procedure.^12^ For these reasons, the possibility of detecting free‐label nanoparticles for in vivo imaging purposes would be certainly a very important objective.

Among the imaging technologies more commonly used in the clinical setting, MRI is likely the more suitable to achieve this goal, because almost any molecular system contains NMR‐detectable atoms,^13^, ^14^, ^15^ and this feature is widely exploited for the direct/indirect MRI detection of diamagnetic probes characterized by the presence of spins with a specific resonance frequency that enables their visualization using spectroscopic imaging approaches, such as Chemical Shift Imaging (CSI).^16^, ^17^, ^18^

CSI is typically used for heteronuclear MRI detection, e.g. for ^19^F‐based agents or hyperpolarized (primarily based on ^3^He, ^13^C, ^15^N, ^129^Xe) probes,^19^, ^20^, ^21^ because for ^1^H detection, the huge signal of water protons, as well as the background signal from the endogenous biomolecules, makes very challenging to get a clear imaging signal from other molecular probes. However, one of the positive consequences of using nanosystems is that, being colloids, they display a high material density, thus offering the opportunity to generate a strong NMR signal that may exceed the tissue background.

This work aims to explore the possibility of detecting, by ^1^H‐MRI, a signal originating from the chemical components of a nanoparticle. A representative example of these chemicals is polyethylene glycol (PEG), a hydrophilic moiety very often chosen to improve the stability and modulate the pharmacokinetic properties of nanosystems, such as micelles and liposomes.^22^ PEG polymer is characterized by a sharp ^1^H NMR signal located ca. 1 ppm upfield from water protons,^23^ which could be detected and monitored, using water suppression pulse sequences, in different acquisition settings such as PRESS, image‐selected in vivo spectroscopy (ISIS), and CSI acquisitions.^24^, ^25^, ^26^, ^27^, ^28^, ^29^, ^30^ The advantages associated with this imaging strategy are manifold. First of all, the nanoparticles do not need to be functionalized with any imaging label. Second, they can be visualized using the standard ^1^H MRI hardware, already available in clinics, without additional costs, and the signal can be monitored in real‐time and quantified through an external reference. In addition, the chemical shift of the components of the nanoparticle is expected to be poorly affected by the tissue environment, with limited variations between the standard reference and the different anatomical regions. Furthermore, the sequences for signal detection do not require the use of additional radiofrequency pulses associated with high specific absorption rate (SAR) values, as necessary for other contrast mechanisms, such as CEST agents.^31^, ^32^, ^33^ A revolutionary applicative aspect is that the signal associated with these molecules can be used to track in vivo the biodistribution of the nanosystem and the delivery of its payload, for example, a drug, without the addition of any further contrast agents, with tremendous applications in the theranostic field.^34^ In addition to PEG, other chemicals can be considered. Koshkina et al. recently reported a method to visualize polymeric micelles using ^31^P MRI, indicating a growing interest in monitoring these systems in real‐time without the need for additional contrast agents.^35^ Intending to test the potential of this approach, in this paper, we propose the use of the following nanosystems successfully used in preclinical and clinical research: stealth lipid‐based micelles and liposomes (in both cases the source of the nanoparticle signal arises from DSPE‐PEG2000),^36^ and perfluorocarbon nanoemulsions (where the detected signal arises from Kolliphor® P188 surfactant).^37^ The latter system has the great advantage of making possible a comparison between ^1^H and ^19^F MR detection. The selected nanoparticles were first characterized in vitro, to assess their detection sensitivity, and then in vivo both on a mouse model of ovarian cancer and of acute neuro‐inflammation. To the best of our knowledge, this is the first piece of research where ^1^H‐CSI detection is proposed to directly visualize free‐label nanoparticles for biomedical imaging applications.

## 2 METHODS

### 2.1 Preparation and characterization of the nanosystems

Three different types of nanosystems were prepared: liposomes, micelles, and PFCE‐NE. More details about the preparation and characterization of the three nanosystems are available in the Data S1.

### 2.2 NMR characterization of the nanosystems

The characterization of the nanosystems was performed at 14T using a high‐resolution NMR spectrometer (see Data S1).

### 2.3 In vitro CSI of the nanosystems

All in vitro MR characterizations of the nanosystems were performed with a 7T MRI scanner (Bruker Avance NEO) equipped with a 40 mm ^1^H/^1^H volume quadrature transmit‐receive probe. For the three phantoms for MRI characterization, six decreasing concentrations (100, 75, 50, 25, 10, 2%) of each nanosystem preparation were diluted 1:1 in 2% low‐gelling agar (final volume 300 μL) and transferred in Eppendorf® Microcentrifuge Tubes. A tube filled with 1% low‐gelling agar was placed in each phantom as a control. Then, the seven tubes were positioned in a 50 mL centrifuge tube filled with double‐distilled water.

After the acquisition of the scout images, and the performance of proper shimming protocols (see Data S1), an anatomical image of the phantom was acquired (RARE, matrix = 256 x 256 pixels, FOV = 30 x 30 mm^2^, spatial resolution = 0.117 x 0.117 mm/pixel, TR = 3 s, TE = 6.19 ms, rare factor (RF) = 16, slice thickness = 1 mm, number of averages (NA) = 2, acquisition time [acq. time]: 1.6 min). Then CSI was performed with the same geometry of the anatomical image, with the following parameters: matrix = 32 x 32 pixels, FOV = 30 x 30 mm^2^, spatial resolution = 0.9375 x 0.9375 mm/pixel, TR = 1 s, TE = 5 ms, NA = 1, FA = π/4, dummy scans (DS) = 2, slice thickness = 1 mm, number of spectral points = 192, SW = 3.97 ppm, spectral resolution = 3.1 Hz/point, o1p = 4.7 ppm, water suppression scheme = VAPOR, water suppression bandwidth = 400 Hz, acq. time = 17 min.

The SNR was used to describe the performance of the method and calculated as SNR=Sns−SagarSDnoise.

The calculation was based on the signal statistics in three separate regions of interest (ROIs) from a single image: one in the region of interest containing the nanosystem to determine the signal intensity ($S_{\mathrm{ns}}$), one in the region containing the blank ($S_{\text{agar}}$), the 1% low‐gelling agar, and one in the image background to measure the statistical intensity distribution of the noise (${\mathrm{SD}}_{\text{noise}}=$ SD of the background intensity).

The limit of detection of this technique was determined by the analysis of the linear dependence of the SNR as a function of the concentration of protons. The limit of detection is defined as the minimum proton concentration significantly different from the blank sample (1% low‐gelling agar).

### 2.4 In vivo CSI of PFCE‐NE—intratumor injection

The in vivo experiments were performed according to the national laws on animal experimentation and approved by the Italian Ministry of Health (Direzione Generale della sanità animale e dei farmaci veterinari) (project identification: 298/2022‐PR, date of approval: 13/05/2022). Mice were kept in standard housing with standard rodent chow and water available ad libitum, and a 12 h light/dark cycle. In order to perform tumor inoculation, PFCE‐NE administration, and imaging, mice (*n* = 3) were anesthetized by intramuscular injection of a combination of 20 mg/kg tiletamine/zolazepam (Zoletil 100; Virbac, Milan, Italy) and 5 mg/kg xylazine (Rompun; Bayer, Milan, Italy). To induce the tumor, 2 x 10^6^ human ovarian carcinoma A2780 cells (ECACC, Salisbury, UK) were suspended in 100 μL of phosphate buffered saline (PBS) and inoculated with a 25G needle into the right flank of two nude athymic female mice of 9 wk of age (Envigo, Gannat, France). When the tumor reached a volume of 350 mm^3^, the animals were recruited. The mice were anesthetized and inserted into a Bruker Pharmascan Avance NEO 7T system with two standard reference tubes, containing 1% low‐gelling agar and PFCE‐NE (proton concentration 2.64 M). After the acquisition of scout images, and the performance of proper shimming protocol (see Data S1) an anatomical image of the tumor was acquired (RARE, Matrix = 128 x 128 pixels, FOV = 30 x 30 mm^2^, spatial resolution = 0.234 x 0.234 mm/pixel, TR = 5 s, TE = 21.6 ms, RF = 8, slice thickness = 1 mm, NA = 2, acq. time: 2.7 min). Then CSI was performed with the same geometry of the anatomical image, with the following parameters: matrix = 32 x 32 pixels, FOV 30 x 30 mm^2^, spatial resolution = 0.9375 x 0.9375 mm/pixel, TR = 1 s, TE = 5 ms, NA = 1, FA = π/4, slice thickness = 1 mm, DS = 2, number of spectral points = 192, SW = 3.97 ppm, spectral resolution = 3.1 Hz/point, o1p = 4.7 ppm, water suppression scheme = VAPOR, water suppression bandwidth = 400 Hz, acq. time = 17 min. At the end of the MRI acquisition, 100 μL of PFCE‐NE (30 mmol protons/kg body weight) were injected directly into the tumor, then the animals were imaged again with the same sequences (the CSI sequence was performed around 20 min post‐injection [p. i.]). A standard reference tube containing PFCE‐NE in low‐gelling agar (2.64 M of protons) was used for proton quantification.

### 2.5 In vivo PRESS of PFCE‐NE—migration to popliteal lymph nodes

In a second in vivo experiment, the in vivo draining of PFCE‐NE into the popliteal lymph nodes (PLNs) was investigated both by CSI and PRESS. One female Balb/C mouse of 8 wk (Envigo) was inoculated with 3 x 10^4^ 4T1 breast cancer cells (ATCC) into the right flank. When the tumor reached a volume of 400 mm^3^ the mouse was recruited. To track the PFCE‐NE in PLNs, the mouse was anesthetized, and then 60 μL (17 mmol protons/kg body weight) of PFCE‐NE were injected into the right footpad. 24 h after the injection the mouse was anesthetized and inserted into the Bruker Pharmascan Avance NEO 7T system. After the acquisition of scout images and of an anatomical image of PLNs (RARE, matrix = 128 x 128 pixels, FOV = 30 x 30 mm^2^, slice thickness = 1 mm, spatial resolution = 0.234 x 0.234 mm/pixel, TR = 5 s, TE = 21.6 ms, RF = 8, NA = 2, acq. time = 2 min 42 s), the proper shim was performed (see Data S1), before the acquisition, on each PLN (left and right) and on each reference tube. PRESS were acquired with the following parameters: voxel size = 1.5 x 1.5 x 1.5 mm^3^, TR = 1.3 s, TE = 16.3 ms, DS = 2, number of spectral points = 2048, SW = 11.1 ppm, NA = 512 for each PLN (acq. Time = 11 min 12 s), while NA = 128 for the reference tubes (acq. time = 2 min 46 s), water suppression scheme = VAPOR, water suppression bandwidth = 400 Hz. Direct CSI acquisition was attempted but was not possible due to bad shimming in the leg area. At the end of the acquisition, the mouse was sacrificed and the PLNs were extracted for ex vivo experiments.

### 2.6 In vivo CSI of PFCE‐NE—intravenous injection

A further in vivo experiment was conducted involving the intravenous injection of PFCE‐NE in a model of acute neuroinflammation (see Data S1). Twenty‐four hours post‐lipopolysaccharides(LPS) administration, PFCE‐NE (50 mmol protons/kg) was intravenously injected. Mice were imaged at 4 and 24 h post‐PFCE‐NE injection using ^1^H CSI. The signal obtained in the right and left striatum, as well as in the surgery area, was measured and quantified using an external reference (317.5 mM ^1^H). More details are reported in the Data S1.

### 2.7 Ex vivo experiments—fluorescence microscopy

At the end of the in vivo MRI experiments, ex vivo experiments by fluorescence microscopy were performed (see Data S1).

### 2.8 Ex vivo experiments—CSI of PLNs

After characterization by fluorescence microscopy, ex vivo experiments by CSI were carried out (see Data S1).

### 2.9 ^19^F MRI vs. ^1^H CSI comparison

As the PFCE‐NE is generally well known for its employment in ^19^F MRI experiments, a comparison between its performance in ^19^F MRI and ^1^H CSI was performed in vitro (see Data S1).

### 2.10 CSI MScript

As the software available for MRI acquisition did not offer a CSI image analysis package, the multi‐voxel spectroscopic images were processed and analyzed using an in‐house script package running in Fiji/ImageJ software. The macros and a brief user guide are provided with this paper (see Supporting information).

### 2.11 Statistical analysis

Data analysis was performed by OriginPro 2016 (64‐bit). Quantitative results were presented as mean values ± SD. GraphPad Prism 8.0.1 was used to generate the graphs.

## 3 RESULTS

### 3.1 DLS Characterization of the nanosystems

To investigate the potential of CSI for detecting free‐label nanosystems, three different types of soft nanoformulations were investigated: phospholipid‐based pegylated micelles (PEG‐MIC) and liposomes (PEG‐LIPO), and perfluorinated nanoemulsions (PFCE‐NE). The formulations displayed a mean size of 14.6 ± 0.4 nm (polydispersity index, PDI, of 0.29 ± 0.06), 119 ± 14 nm (PDI 0.09 ± 0.03), 135 ± 15 nm (PDI 0.12 ± 0.04), respectively. The mean particle concentration was estimated to be around 3.87 x 10^15^ micelles/mL, 1.85 x 10^14^ liposomes/mL, and 7.77 x 10^14^ NPs/mL for PEG‐MIC, PEG‐LIPO and PFCE‐NE, respectively.

### 3.2 NMR Characterization of the nanosystems

**FIGURE 1:**
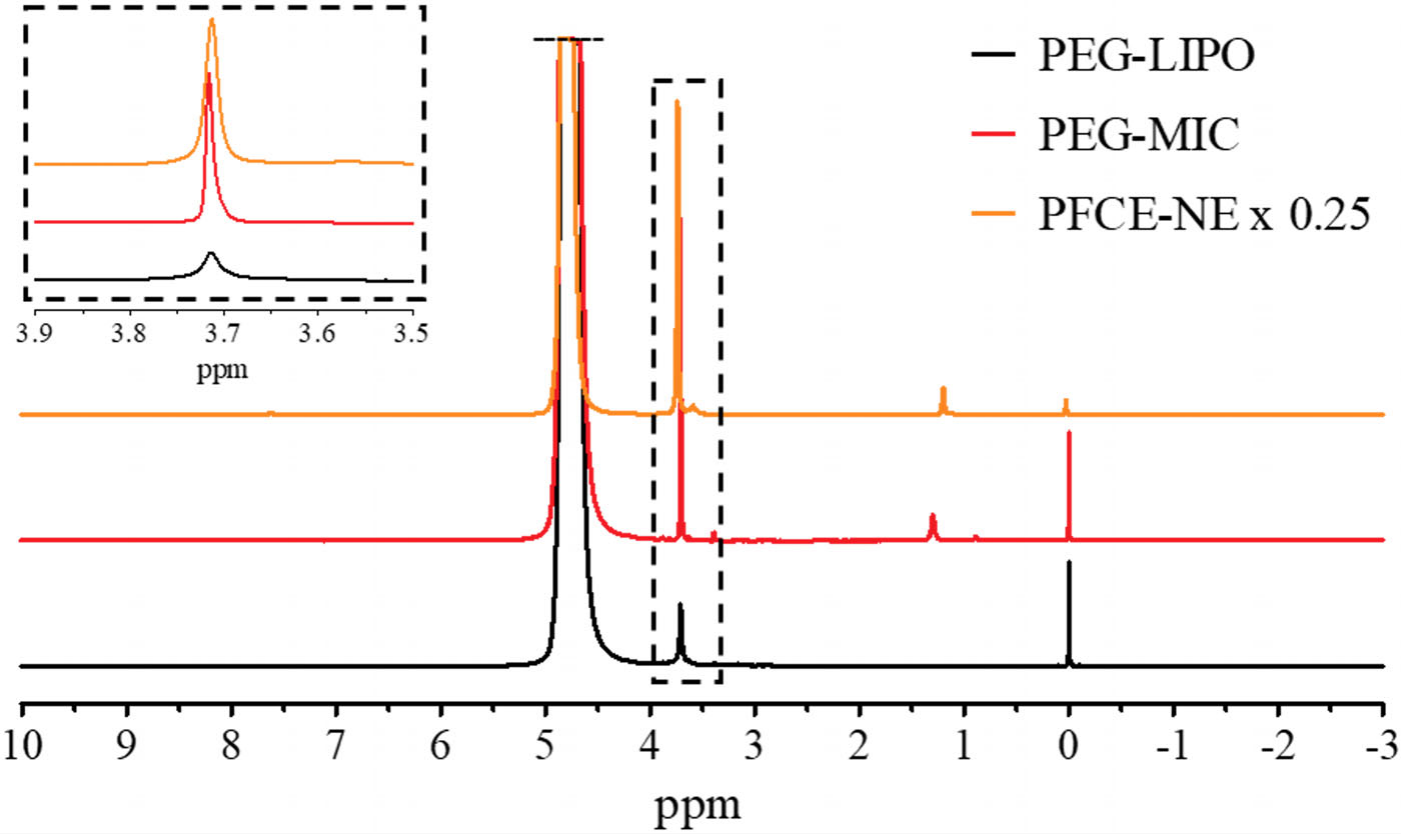
High‐resolution NMR spectra of (PEG‐LIPO), PEG‐MIC, and PFCE‐NE (diluted 1:4). High‐resolution NMR spectra of PEG‐LIPO (black line), PEG‐MIC (red line), and PFCE‐NE (orange line) samples acquired at 14T (298 K), without water suppression. A magnification of the 3.5–3.9 ppm spectral window is displayed in the dashed frame, to better appreciate the main peak of DSPE‐PEG2000 and Kolliphor® P188 (3.71 ppm). The peak at 0 ppm corresponds to TSP‐d_4_, used for proton quantification. The assignment of the other visible peaks is reported in Figures S1 and S2. The PFCE‐NE spectrum intensity has been reduced to the 25% of the original value, for comparison purposes.

First, a high‐resolution NMR spectrum was acquired at 14T, revealing the presence of a single well‐defined peak at 3.7 ppm for DSPE‐PEG2000 and Kolliphor® P188, with additional smaller peaks in the 1–3.5 ppm region (Figure 1 and Figures S1 and S2). During the acquisition of these spectra, the water signal was not suppressed to avoid the accidental suppression of the nanosystem‐associated signal. The mean proton concentration was determined for the three nanosystems, through an internal standard reference (TSP‐d_4_). PFCE‐NE displayed the highest proton concentration (6.35 M of protons), while the concentration of DSPE‐PEG2000‐associated protons in PEG‐MIC and PEG‐LIPO suspension was comparable: 0.99 and 0.57 M, respectively (Table 1). The longitudinal (T_1_) and transversal (T_2_) relaxation times of such protons were measured at the same magnetic field and two temperatures: 298 and 310 K (Table 1). The T_1_ values at 298 K ranged from 0.6 to 0.7 s with an expected, slight, lengthening at 310 K. The T_2_ decays are faster, especially for PEG‐LIPO, where a bi‐exponential behavior was observed.

**TABLE 1:** Values of proton concentrations, and longitudinal and transversal relaxation times (measured at 298 K and 310 K) determined for each nanosystem at 14T.

Nanosystem type	Mean protons concentration (M of protons)	Relaxation time (s)	298 K	310 K
PEG‐LIPO	0.57	T_1_	0.64 ± 0.02	0.87 ± 0.05
		T_2_	0.03 ± 0.01	0.04 ± 0.02
			0.12 ± 0.03	0.26 ± 0.08
PEG‐MIC	0.99	T_1_	0.716 ± 0.002	0.88 ± 0.02
		T_2_	0.453 ± 0.005	0.602 ± 0.009
PFCE‐NE	6.35	T_1_	0.775 ± 0.006	0.975 ± 0.008
		T_2_	0.383 ± 0.007	0.201 ± 0.003

### 3.3 In vitro CSI of the nanosystems

**FIGURE 2:**
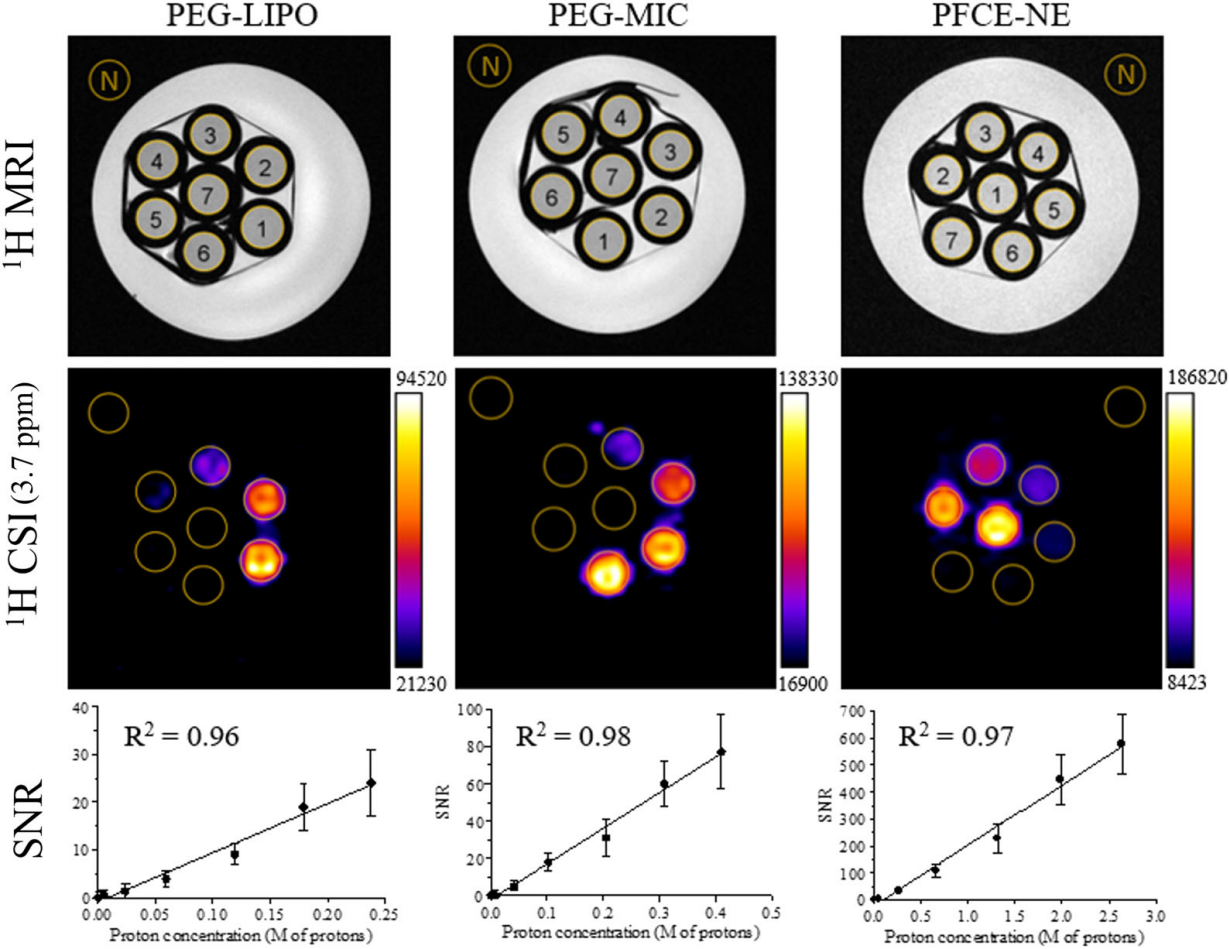
In vitro ^*1*^H‐CSI of PEG‐LIPO, PEG‐MIC, and PFCE‐NE. In vitro MRI of phantoms containing PEG‐LIPO, PEG‐MIC, and PFCE‐NE samples diluted at different concentrations in low‐gelling agar (sample 1 corresponds to the highest concentration of each nanosystem: 0.240 M ^1^H and 7.7 x 10^13^ liposomes/mL for PEG‐LIPO, 0.410 M ^1^H and 1.6 x 10^15^ micelles/mL for PEG‐MIC, 2.640 M ^1^H and 3.2 x 10^14^ NPs/mL for PFCE‐NE, sample 6 to the lowest one: 0.005 M ^1^H and 1.5 x 10^12^ liposomes/mL for PEG‐LIPO, 0.008 M ^1^H and 3.2 x 10^13^ micelles/mL for PEG‐MIC, 0.050 M ^1^H and 6.5 x 10^12^ NPs/mL for PFCE‐NE, sample 7 to pure low‐gelling agar). MRI was performed at 7T (Bruker Avance) with a quadrature coil. The mean SNR values calculated over the external noise (N) are displayed for each nanosystem. The error bars correspond to the SD of the SNR in each ROI.

To investigate the imaging potential, a phantom was prepared for each nanosystem, containing serial dilutions of the nanocarriers in agar gel. The phantom was then positioned into a 7T Bruker MRI scanner equipped with a microimaging probe and imaged by ^1^H‐CSI (Figure 2). Once a proper shim protocol was executed (see Data S1), the linewidth values obtained for the peak at 3.8 ppm in the phantoms were measured to be between 15 and 24 Hz.

The highest SNR value (580 ± 110) was observed for the PFCE‐NE sample, as expected, due to the higher proton concentration of this sample. PEG‐LIPO and PEG‐MIC samples displayed similar values, in line with their proton concentration. Remarkably, at the same concentration of protons, the SNR of PEG‐LIPO was half of that observed for PEG‐MIC. This discrepancy may be attributable to the broader signal for this sample, which can be associated with the shorter T_2_ value. The results obtained showed excellent linearity between the proton concentration (correlated to the amount of nanoparticles) and the MRI response. The use of a relatively short TR (TR = 1 s) in the sequence was a relevant prerequisite for reducing the duration of the overall acquisition (17 min for the experiments reported in Figure 2). Any effect due to the partial magnetization recovery between the sequence pulses was attenuated by adding two dummy scans before each acquisition.

### 3.4 In vivo CSI of PFCE‐NE–intratumor injection

**FIGURE 3:**
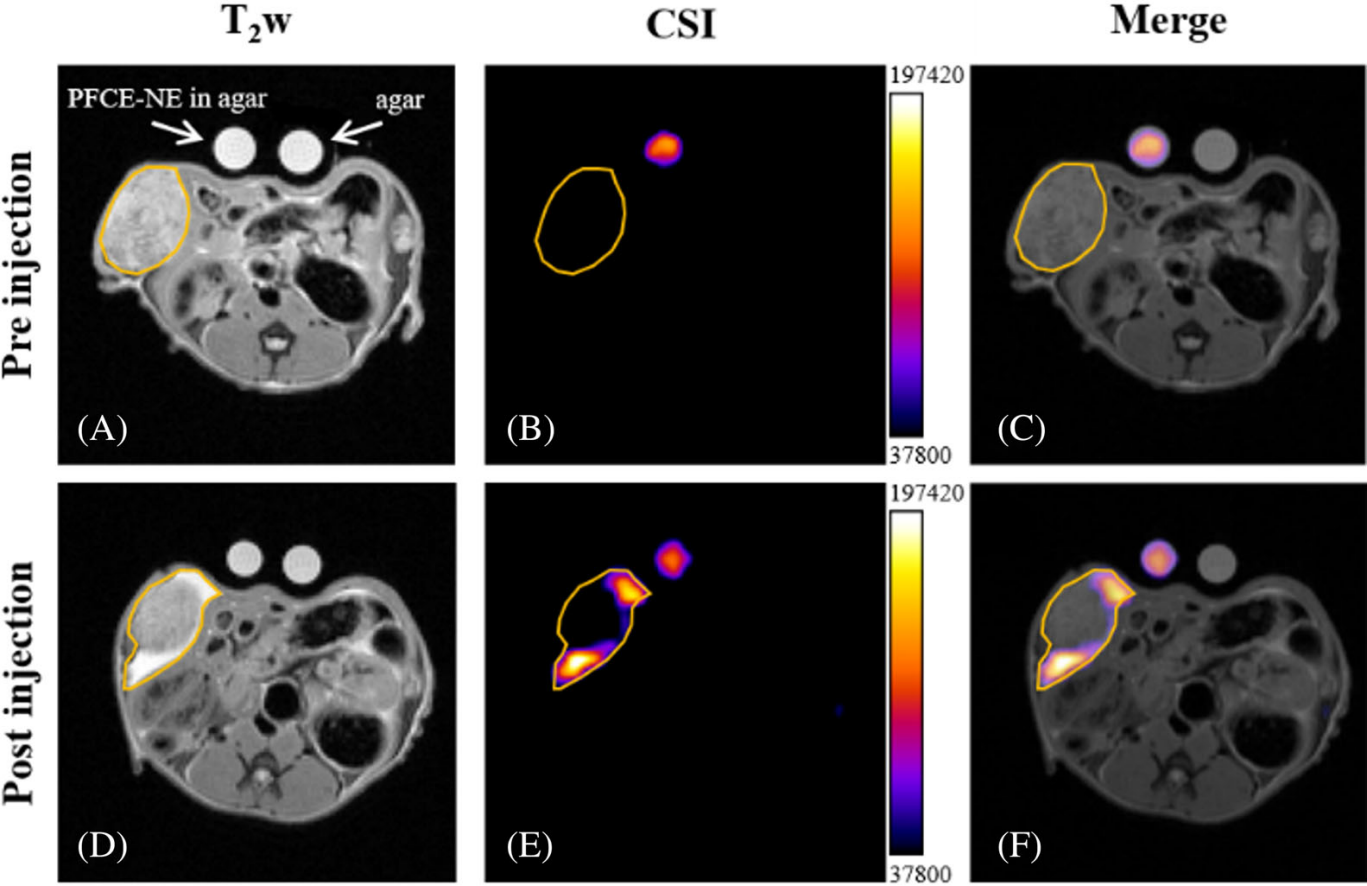
In vivo CSI after intratumoral injection of PFCE‐NE. In vivo MRI (7T, Bruker Pharmascan) of a nude mouse bearing a subcutaneous ovarian cancer (A2780 cell line) in the right flank. Anatomical T_2_ weighted images of the tumor are displayed before (A) and 10 min after (D) the intratumoral injection of the PFCE‐NE (100 μL, corresponding to around 30 mmol protons/kg body weight). The CSI of the same slices, at 3.71 ppm, was performed before (B) and 30 min after (E) the intratumoral injection of the PFCE‐NE. In (C) and (F) the merging of the T_2_ weighted and CSI images is reported. The two circles placed in front of the mouse correspond to two different standard reference tubes: The left tube contains the PFCE‐NE in agar (proton concentration 2.64 M), and the right tube corresponds to pure low‐gelling agar.

Moving to the first in vivo test, a small volume (100 μL) of a suspension of PFCE‐NE (corresponding to 30 mmol protons/kg body weight) was administered directly into a tumor induced by the subcutaneous inoculation of human ovarian carcinoma cells (A2780) on immunocompromised mice (Figure 3). CSI images acquired before the injection of the nanoparticles demonstrated the absence of any signal at the target frequency (3.7 ppm) (Figure 3B,C), while 30 min after the injection of the nanoemulsion, a clear signal was detected in the tumor rim, both by classical T_2_ weighted MRI (Figure 3D), which reports about the liquid accumulation, and by ^1^H‐CSI (Figure 3E,F). The standard reference tube of PFCE‐NE placed next to the mice allowed not only for unambiguous identification of the signal but also for direct quantification of the amount of the PFCE‐NE‐associated protons located in the tumor area. In particular, in the representative slice reported in Figure 3, the amount of PFCE‐NE protons corresponded to 9% of the injected dose (0.057 mmol), a reasonable value considering that the imaged slice represented ca. 10% of the whole lesion. The mean number of PFCE‐NE protons found in a single slice in the three mice examined corresponded to 0.063 ± 0.007 mmol, Table S1.

### 3.5 In vivo PRESS of PFCE‐NE–migration to PLNs

**FIGURE 4:**
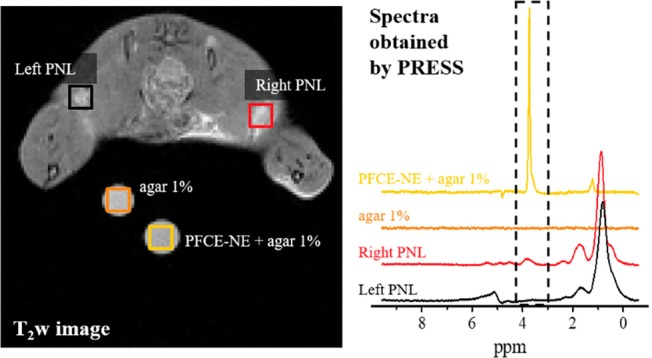
In vivo PRESS of PLNs acquired 24 h post PFCE‐NE injection in the footpad. In vivo MRI and PRESS of the PLNs of a 4T1 tumor‐bearing mouse performed at 7T (Bruker Pharmascan). Left: Anatomical T_2_ weighted image of the PLNs. The two circles placed in front of the mouse correspond to the two standard reference tubes. The squares pictured in the image correspond to the voxels acquired by PRESS: Black, PLN of the left leg; red, PLN of the right leg (tumor‐bearing side); orange, pure low‐gelling agar; yellow, PFCE‐NE in low‐gelling agar (0.66 M proton concentration). Right: Spectra obtained by PRESS in the 0 to 10 ppm spectral region are reported. The water signal was suppressed. The peak corresponding to PFCE‐NE is highlighted with a dashed line.

Next, a second, and more clinically relevant, in vivo proof of concept was performed by injecting PFCE‐NE (17 mmol protons/kg bw) into the right footpad of mice bearing (in the same mouse side) a subcutaneous murine breast cancer (induced using 4T1 cells) to monitor the recruitment of the nanoparticles by the immune system and follow their migration and accumulation in PLNs.^38^ At 24 h post‐administration, mice were subjected to a ^1^H‐CSI session. Due to the presence of various interfaces between the mouse body, the reference tube, and the empty space of the coil, the better shimming conditions achieved did not allow good water suppression, thus preventing the ^1^H‐CSI detection. Therefore, in this case, a localized shim protocol was applied (see Data S1), and the PFCE‐NE signal was detected on the two PLNs and the two reference tubes (for quantification) by using PRESS. The resulting spectra clearly demonstrated the presence of the nanoparticles only in the right PLN corresponding to the mouse side where the tumor lesion was located (Figure 4).

The peaks observed in both the PLNs in the 0–2 ppm regions corresponded to the signal of the lipidic components surrounding the PLNs. Quantification of the proton signal using the external reference indicated that 0.33 μmol of PFCE‐NE‐associated protons were localized into the right PLN, corresponding to around 0.1% of the injected dose. As expected, the nanoemulsion was drained to the PLN through the lymphatic pathway.^39^ From the analysis of the AUC obtained from the PRESS acquisitions, and comparing them with the reference tubes, a proton concentration of 98 mM was calculated for the right PLN.

### 3.6 Ex vivo experiments–CSI of PLNs and fluorescence microscopy

**FIGURE 5:**
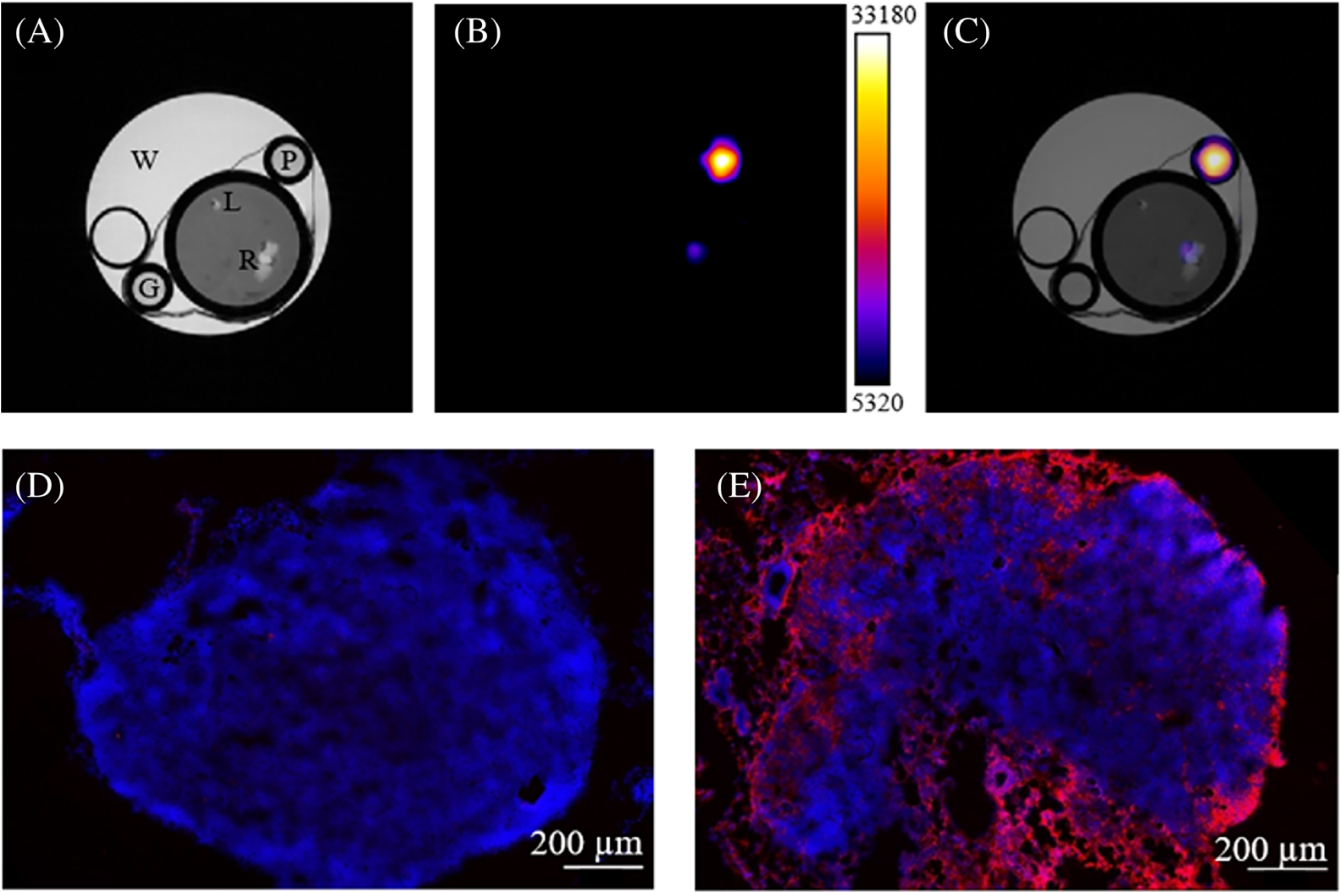
Ex vivo ^1^H‐CSI and confocal fluorescence microscopy of PLNs after PFCE‐NE injection in the footpad. Ex vivo MRI of the PLNs of a 4T1 tumor‐bearing mouse performed at 7T. In (A) the anatomical T_2_ weighted image of the PLNs is reported. In (B) the CSI‐related signal, acquired at 3.7 ppm, is displayed. In (C) merging of (A) and (B) is performed to perfectly localize the signal. R corresponds to the right PLN, L to the left PLN, G to a standard reference tube containing pure low‐gelling agar, P to a standard reference tube containing PFCE‐NE in low‐gelling agar (proton concentration 0.66 M), W to a water containing tube. In (D, E) confocal microscopy images of (D) the left PLN and (E) the right one are displayed: Blue corresponds to nuclear staining (DAPI), while red corresponds to the rhodamine signal (PFCE‐NE). The scale bar corresponds to 200 μm.

At the end of the MR acquisitions, the animal was sacrificed and the PLNs were excised for ex vivo validations. An agar phantom containing two reference tubes and the PLNs was prepared and immediately acquired by ^1^H‐CSI. No fixation of the sample was performed to avoid signal alteration by formalin addition. The homogeneous composition of the phantom allowed for finding a good shimming condition for CSI detection (32 x 32 matrix, total acq. time 17 min). In these conditions, only the signal from the right PLN was clearly detected (Figure 5). Signal quantification through the external reference (0.66 M of protons), led to an amount of PFCE‐NE‐associated protons (0.26 μmol) very close to the value determined by PRESS detection (0.33 μmol), thus confirming the excellent quantification potential of the technique. The difference in PLN size, clearly visible in Figure 5A, is due to the PLN enlargement induced by the tumor.^40^ The ex vivo confocal fluorescence microscopy proved the presence of the PFCE‐NE (doped with a phospholipid conjugated with rhodamine) mainly in the subcapsular region of the right PLN (Figure 5E). In the left PLN (Figure 5D), a few different red spots can be also visualized, suggesting the lymphatic drainage also into the contralateral leg, however, the amount of PFCE‐NE was too low to be detected by ^1^H CSI.

Hematoxylin and eosin staining of the PLNs were also performed and are reported in Figure S3.

### 3.7 In vivo CSI of PFCE‐NE–intravenous injection

**FIGURE 6:**
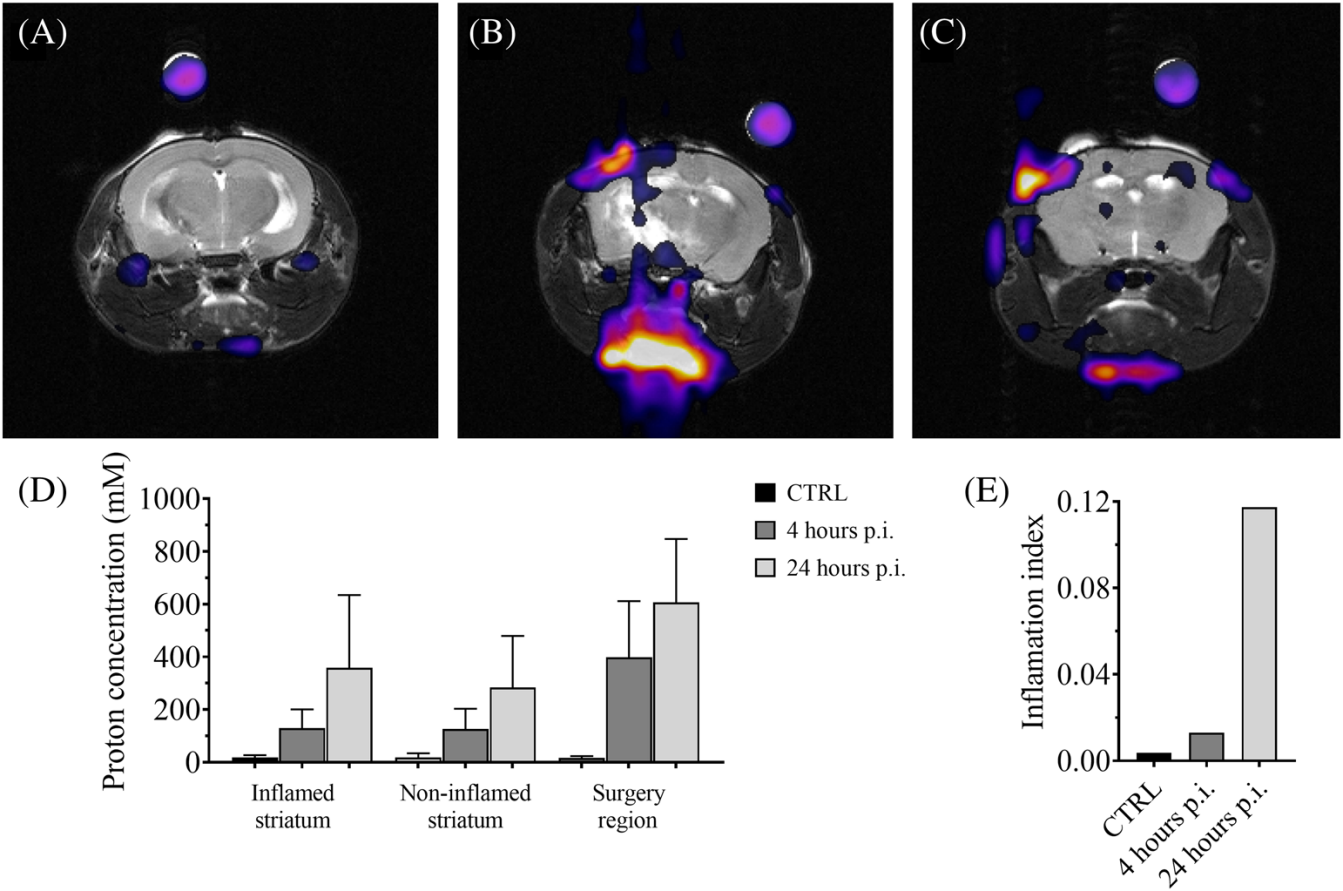
In vivo ^1^H CSI after intravenous injection of PFCE‐NE in a murine model of inflammation. In vivo MRI (7T, Bruker Pharmascan) images of a murine model of inflammation. Anatomical T_2_‐weighted images overlaid with CSI images at 3.71 ppm are shown in the top part of the figure: (A) before, (B) 4, and (C) 24 h post iv injection of PFCE‐NE (50 mmol protons/kg bw). The bottom part of the figure displays graphs showing the (D) quantitative results of PFCE‐NE proton concentration in the two regions of the striatum, both inflamed and non‐inflamed, as well as in the surgery region, and (E) the inflammation index, indicating the asymmetric accumulation of the nanosystem between the inflamed and non‐inflamed striatum.

A further and more relevant in vivo experiment was conducted on a murine model of inflammation. LPS was injected into the right striatum of five mice to induce inflammation. In three of these mice, 50 mmol protons/kg body weight of PFCE‐NE were injected into the tail vein. CSI images were acquired before administration and then at 4 and 24 h after the systemic injection of the nanosystem (Figure 6A–C). In the control images obtained before the PFCE‐NE administration, no detectable signal was observed at the specific frequency of our nanosystem. While, at 4 and 24 h p. i., an increasing accumulation of nanoparticles can be observed in the inflamed areas, recognizable at the LPS injection site due to the surgical action, as well as in the striatal areas where inflammation was induced. The presence of the nanosystem was more pronounced in the regions affected by the surgery, a known effect observed in previous studies.^41^ Nevertheless, there was a marked increase in signal in the striatum at 4 and then at 24 h p. i., with a slightly higher accumulation in the hemisphere associated with the inflammation (Figure 6D). To investigate region‐specific increases in PFCE‐NE uptake into the inflamed area, an inflammation index was defined to show the asymmetry in the nanosystem accumulation in the inflamed region (Figure 6E). Moreover, the images (Figure 6B,C) show a strong signal corresponding to the blood vessels in the lower part of the mouse's head, as expected since the half‐life time of the PFCE‐NE in the blood is around 11.5 h.^37^

### 3.8 ^19^F MRI vs. ^1^H CSI comparison

**FIGURE 7:**
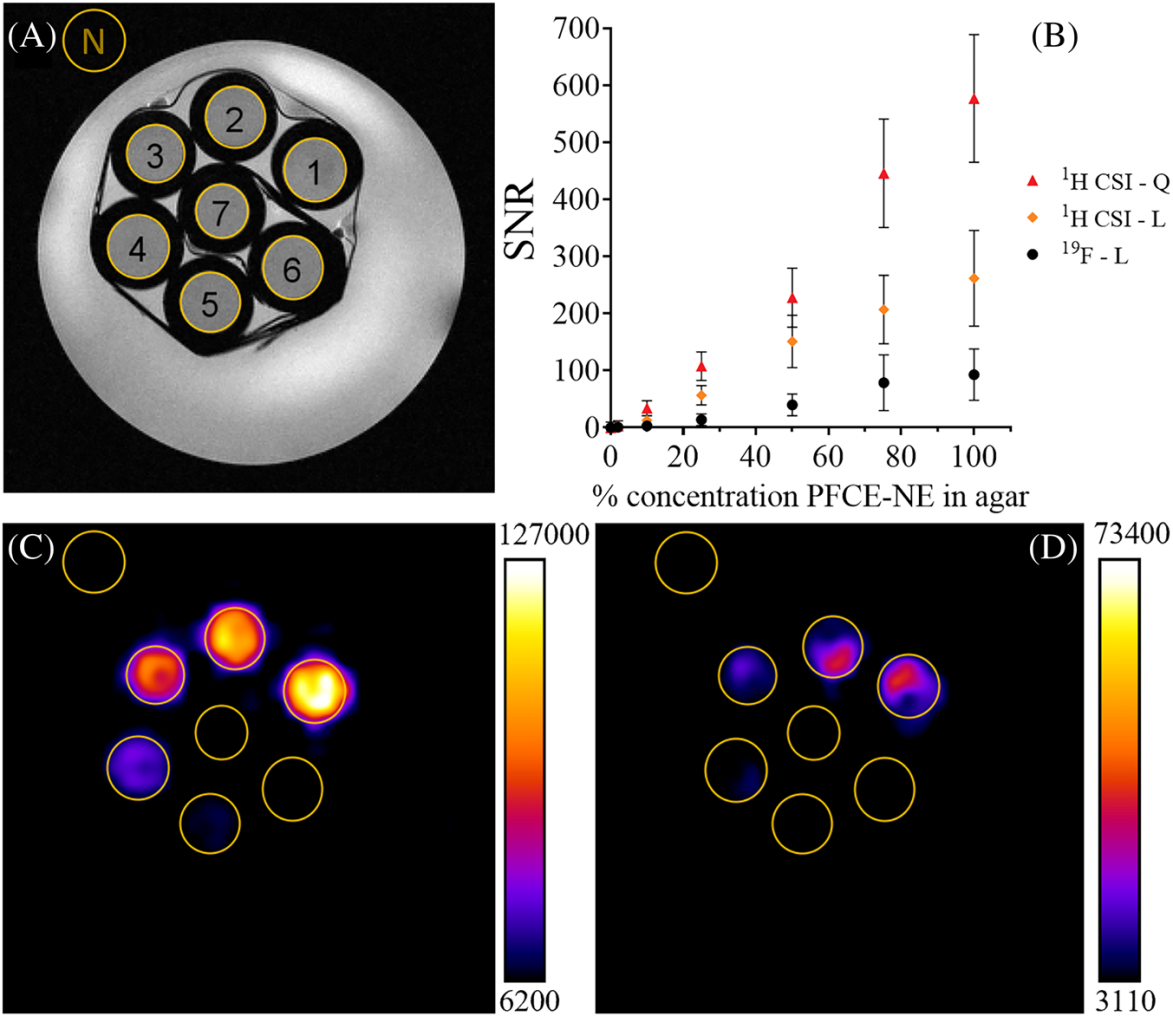
Comparison between ^1^H CSI and ^19^F MRI detection modes for PFCE‐NE. Comparison between ^1^H CSI and ^19^F MRI carried out at 7T (Bruker Avance 300 MHz) with a 40 mm ^1^H/^19^F volume transmit‐receive probe. (A) T_2_ weighted image of the phantom, containing different dilutions of PFCE‐NE in low‐gelling agar (sample 1 corresponds to the highest concentration of PFCE‐NE: 6.35 M ^1^H, 3.2 M ^19^F, and 7.77 x 10^14^ NPs/mL, sample 6 to the lowest one: 0.130 M ^1^H, 0.064 M ^19^F, and 1.55 x 10^13^ NPs/mL, sample 7 to pure low‐gelling agar). (B) Comparison between the SNR values calculated over the external noise (N) for ^1^H CSI acquired with a quadrature coil (red triangles), ^1^H CSI acquired with a linear ^1^H/^19^F coil (orange diamonds), or ^19^F MRI (black circles). On the x‐axis, the % dilution of PFCE‐NE in low‐gelling agar is reported to better compare the two imaging techniques. In (C), the ^1^H CSI of the phantom acquired with the 40 mm ^1^H/^19^F volume transmit‐receive probe, at 3.71 ppm is displayed. In (D), the ^19^F MRI of the phantom is reported.

Finally, being PFCE‐NE a contrast agent usually employed for ^19^F MRI detection, it was deemed interesting to make a direct comparison between ^1^H‐CSI and ^19^F‐MRI detection modes (Figure 7).

To this purpose, the same phantom prepared for the ^1^H‐CSI detection of PFCE‐NE was subjected to ^19^F MRI. As the ^1^H/^19^F T_1_ values of Kolliphor® P188 (^1^H) and PFCE (^19^F) are very close (ca. 0.8 s),^37^ the same TR value of 1 s was used in the pulse sequences. Moreover, the same matrix size (32 x 32) and acq. time (17 min) were used for the two nuclei. Importantly, the results obtained demonstrated higher sensitivity for ^1^H CSI detection, with a three‐fold increase in the SNR value, mainly justified by the higher concentration in protons than fluorine atoms for the nanoemulsion (6.35 M vs. 3.2 M, respectively). The higher sensitivity of the CSI approach was tested with a linear coil to directly compare the two techniques. However, as reported in Figure 2C, when using a quadrature coil, the efficiency of the CSI was even higher than ^19^F MRI (with the quadrature coil the SNR enhanced from 260 ± 80 to 580 ± 110 for the same sample) (Figure 7B).

## 4 DISCUSSION

To investigate the potential of CSI for detecting free‐label nanosystems, three different types of soft nanoformulations were investigated: micelles, liposomes, and perfluorinated nanoemulsions. Among these preparations, liposomes are the only ones routinely employed in clinics. The PEG‐LIPO formulation here investigated is a Doxil‐like formulation,^42^ but with a higher percentage of the pegylated component DSPE‐PEG2000 (10% instead of 5%), which is the proton source for the CSI detection. Instead, pegylated micelles and PFCE‐NE have been widely employed and investigated in preclinical and clinical studies.^37^, ^41^, ^43^, ^44^, ^45^, ^46^ The latter, in particular, are generally used for detecting the ^19^F MRI signal in cellular imaging applications, while in this work, the attention was focused on its proton‐rich surfactant component.

The three samples were prepared to get a final nanoparticle concentration compatible with the in vivo injected doses for the corresponding systems. Following size characterization, the high‐resolution NMR spectra were acquired at 14T for the three nanosystems, showing a single well‐defined peak at 3.7 ppm for DSPE‐PEG2000 and Kolliphor® P188, with smaller peaks in the 1–3.5 ppm range. In terms of proton concentration, PFCE‐NE displayed the highest concentration (6.35 M of protons, corresponding to 7.77 x 10^14^ NPs/ml), while the concentration of DSPE‐PEG2000‐associated protons in PEG‐MIC and PEG‐LIPO suspension was lower (0.99 and 0.57 M, respectively). T_1_ and T_2_ relaxation times were measured at 298 and 310 K, ranging from 0.6 to 0.7 s for T_1_ and showing faster T_2_ decays, particularly for PEG‐LIPO, where a bi‐exponential behavior was observed, likely somehow attributable to the presence of two dynamically different environments for the polymer: the one pointing inward to the aqueous core of the nanovesicle, and the other pointing outward and exposed to the bulk solvent. The favorable characteristics of the high‐resolution NMR spectra in terms of signal intensity and frequency selectivity prompted us to explore the possibility of visualizing the free‐label nanosystems by ^1^H‐CSI. Of course, due to the vicinity of the water peak (4.7 ppm), a good shim and an effective water suppression protocol are required (see Data S1), as normally demanded by most MR spectroscopic protocols. To investigate the imaging potential, a phantom was prepared for each nanosystem, containing serial dilutions of the nanocarriers in agar gel. ^1^H‐CSI was selected as the technique of choice due to a series of advantages. First of all, it offers the chance to observe a precise molecule, endogenous or exogenous, based on the specific chemical shift of its NMR signal. Then, the multi‐voxel spectroscopy that utilizes phase‐encoding for spatial localization, allows for obtaining molecular maps through MRI. Finally, the potential for a wide coverage area allows for the evaluation of the signal, and therefore the molecule of interest, in heterogeneous samples, such as living organisms. On the other side, the major disadvantages of multi‐voxel CSI include the long set‐up and imaging time, which are the most critical constraints of this kind of acquisition, and the difficulties in obtaining homogenous shim over the entire region of interest. Various strategies for reducing scan time, however, are potentially available, such as echo‐planar spectroscopic imaging (EPSI), which samples only a limited region of k‐space, or frequency‐selective sequences for molecular imaging (chemical shift selective imaging [CSSI]).^47^ In the in vitro experiments performed the highest SNR value was observed for the PFCE‐NE sample, probably due to its higher proton concentration. The in vitro detection limit (with the current experimental setup), in fact, was around 50 mM of protons for all the samples, corresponding to a nanosystem dilution of around 12, 20, and 130 times, for PEG‐LIPO, PEG‐MIC, and PFCE‐NE, respectively, and to a nanoparticle concentration of around 1.6 x 10^13^ liposomes/mL, 1.9 x 10^14^ micelles/mL and 6.1 x 10^12^ NPs/mL for PEG‐LIPO, PEG‐MIC, and PFCE‐NE, respectively. Since after the intravenous administration in a mouse, a nanosystem is immediately diluted in the blood by ca. a 1:14 factor (calculated considering an injected volume of 125 μL and total blood volume of 1.8 mL),^48^ the PFCE‐NE turned out to be the most promising system for attaining an in vivo proof of concept of this approach. As the CSI signal is directly proportional to the concentration of PEG, it is expected that by decreasing the concentration of PEG while keeping all other phospholipids constant, the SNR will proportionally decrease. For instance, reducing the PEG concentration from 10% to 5% is expected to halve the SNR. Furthermore, the length of the PEG chain surely influences the CSI signal, with higher SNR expected for longer PEG chains (e.g., PEG5000‐6000). However, variations in PEG chain length may also affect T_1_ and T_2_ relaxation times, which requires further investigation. Since PEG 2000 is the most commonly used PEGylated phospholipid in the preparation of nanoparticles, our focus has been on this specific PEG chain length.

The first demonstration of possible in vivo application of ^1^H‐CSI using PFCE‐NE was obtained by direct intratumor injection of PFCE‐NE followed by ^1^H‐CSI of the tumor, displaying a clear accumulation of the nanoemulsion in the tumor rim. This accumulation was already visible by T_2w_ MRI, but ^1^HCSI provided also quantitative analysis. The quantitative potential of this approach, indeed, is extremely intriguing as quantification is not easily accessible for classical T_1_ and T_2_ MRI detection of imaging probes, where the observed signal is not directly associated with the amount of contrast agent, but with the effect exerted by the contrast agent on the nearby water molecules.^49^ However, we are aware that the intratumor injection of NPs is seldom used, limited to brain tumor applications, therefore ^1^H CSI following systemic administration would be more interesting. In a second experiment, PFCE‐NE was injected into the footpad of mice bearing subcutaneous murine breast cancer, with subsequent monitoring of migration and accumulation in PLNs. Unfortunately, in this case, the CSI was not successful due to bad shimming conditions, however, it was possible to monitor and quantify the proton signal in PLNs utilizing localized shim protocol and PRESS. More in detail, the PFCE‐NE accumulation was observed specifically in the right PLN, corresponding to the tumor location. The quantification of PFCE‐NE protons in the right PLN revealed drainage through the lymphatic pathway, accounting for approximately 0.1% of the injected dose. Ex vivo validation through CSI of excised PLNs confirmed the presence of PFCE‐NE predominantly in the right PLN, consistent with in vivo observations and most importantly, the quantification of PFCE‐NE protons using external reference tubes demonstrated agreement with in vivo quantification methods, highlighting the technique's reproducibility and reliability. At the same time, this result highlights the absolute need for a perfect shimming to proceed with the CSI acquisition. In fact, the incomplete saturation of water can lead to the presence of multiple artifacts at various frequencies, thus making difficult the unambiguous identification of the nanoparticle signal or even the visualization of the signal itself. Considering the results obtained, the final and most intriguing in vivo experiment was conducted using a model of acute neuroinflammation. In this experiment, PFCE‐NE was injected directly intravenously, and the signal was detected and quantified in the brain before and at 4 and 24 h p. i. This demonstrated, for the first time, the technique's ability to visualize NP accumulation following systemic administration. No significant signal was detected prior to the PFCE‐NE injection. However, post‐injection, the NPs were detectable in the striatum, blood vessels, and especially at the surgery site, due to the intense inflammation associated with the operation. Observing an anatomical region like the brain ensured optimal shimming conditions. These findings suggest the potential for visualizing other nanosystem targeting, particularly in tumors, which is highly promising for theranostic applications. Nonetheless, further investigation is required to fully optimize the shimming and acquisition parameters. At the current state, we do not expect that Doxil can be visualized in tumors, but with optimization of the technique, this might become possible. Finally, as in the PFCE‐NE emulsion both ^1^H and ^19^F nuclei are present, a direct comparison was made between the ^1^H CSI and ^19^F MRI performance. The results obtained revealed a superior sensitivity in ^1^H CSI detection, with a three‐fold increase in the SNR value. This enhancement can be primarily attributed to the higher concentration of protons compared to fluorine atoms in the nanoemulsion. To further validate the heightened sensitivity of the CSI approach, a direct comparison was conducted using a linear coil. However, when employing a quadrature coil, the efficiency of CSI surpassed that of linear ^19^F MRI, highlighting the extensive potential of the proposed technique.

## 5 CONCLUSIONS

In summary, this work proposes, for the first time, an imaging methodology for the in vivo ^1^H‐CSI detection of free‐label nanoparticles of pre‐clinical and clinical relevance in biomedicine. The results here presented look promising and prompt for further investigations to better assess the real potential of this approach, because, in principle, all the biomedical research areas that involve nanotechnology could receive great benefits. Currently, the main weak points are represented by the need to have an optimal magnetic field homogeneity, a high detection sensitivity, and a reduction of the scanning times, but all these limitations could be overcome by hardware and chemical optimization, thus paving the way to the exploration of innovative applications.

## FUNDING INFORMATION

This research was funded by Associazione Italiana per la Ricerca sul Cancro (AIRC, IG‐22041) and by the University of Torino (Ricerca Locale 2021). The funding source has no involvement in study design, in the collection, analysis, and interpretation of data, in the writing of the report, and in the decision to submit the article for publication.

## CONFLICT OF INTEREST STATEMENT

The authors disclose the filing of an Italian patent entitled “Nanosystems comprising label‐free nanoparticles and uses thereof” concerning the topic of this manuscript (application date: 08/08/2023, N° 102023000016998).

## Supporting information


**Data S1.** Methods: Preparation and characterization of the nanosystems; NMR characterization of the nanosystems; Shim protocol; Ex vivo experiments–Fluorescence microscopy; Ex vivo experiments–CSI of PLNs; In vivo CSI of PFCE‐NE–intravenous injection; ^19^F MRI vs. ^1^H CSI comparison.
**Figure S1.** High‐resolution NMR spectra of DSPE‐PEG2000 in liposomes and micelles.
**Figure S2.** High‐resolution NMR spectrum of Kolliphor® P188 in PFCE‐NE.
**Table S1.** The measured SNR, the mean number of μmol of protons found in a single tumor slice (1 mm of thickness), and the % of moles of protons found in the tumor slice over the total injected proton moles are reported (*n* = 3).
**Figure S3.** Hematoxylin and Eosin staining of PLNs.
