# Supplementary material for: MRI detection of free‐contrast agent nanoparticles
Source: Magn Reson Med. 2024 Sep 29;93(2):761–74. doi: 10.1002/mrm.30292 (PMC11604830; doi:10.1002/mrm.30292)

Guidelines for Processing and Visualizing CSI MRI Images in ImageJ - Fiji: Macros for PV 360 Data

This documentation details three macros designed for processing and visualizing CSI MRI images acquired using the PV 360 software on ImageJ - Fiji.

These macros facilitate the handling of CSI data, including the visualization of NMR spectra and the integration of frequency-specific signals. Each script includes indications within the code to guide the user on which sections may require modification.

Macro 1: 1_CSI_Specta_EC

The 1_CSI_Spectra_EC macro is designed to open CSI images as a stack of two-dimensional images, where each slice corresponds to the acquired pixel matrix and the number of slices equals the number of points in the NMR spectrum.

Additionally, the macro activates a tool (bottom right of the image) that enables real-time visualization of the NMR spectrum within a user-defined region of interest (ROI). This spectrum updates automatically when the ROI is adjusted, provided that the "Live" button is activated in the "Reslice" window.

Individual spectra can be exported to a .csv file through the "Reslice" window by navigating to Data -> Save data.

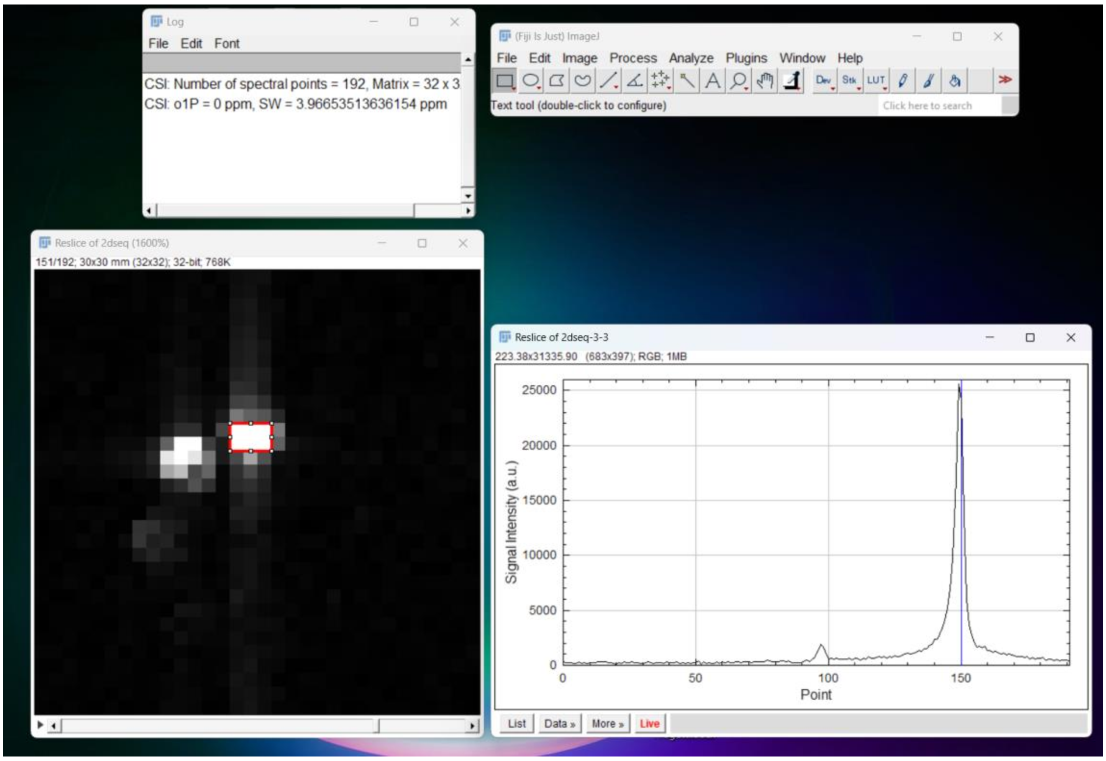

Macro 2: 2_CSI_FreqMap_EC

The 2_CSI_FreqMap_EC macro allows the user to generate an image by integrating the signal for each pixel within a selected frequency range.

For example, in this test, an image is created from the signal between spectral points 147 and 158, which correspond to the extremities of the PFCE-NE signal at 3.8 ppm.

The script produces two images: one without interpolation and another with a blur effect akin to that in PV360 tools. Both images are scaled and translated to align with the matrix of the anatomical MRI image (e.g., T_2-weighted image) that will be opened using the next script.

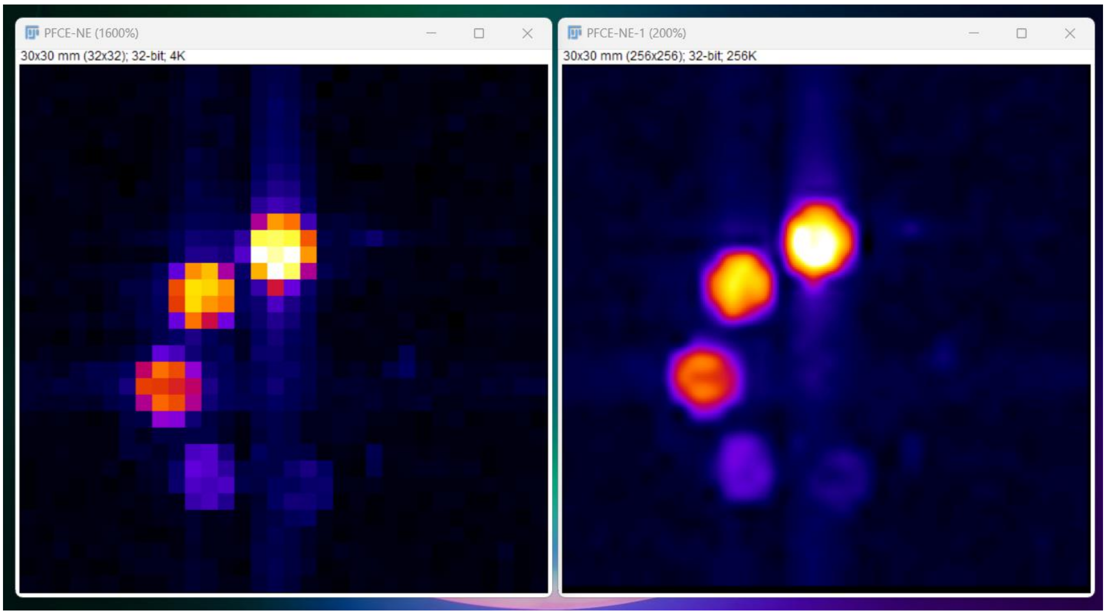

Macro 3: 3_Anatomical_EC

The 3_Anatomical_EC macro opens the reference anatomical MRI image.

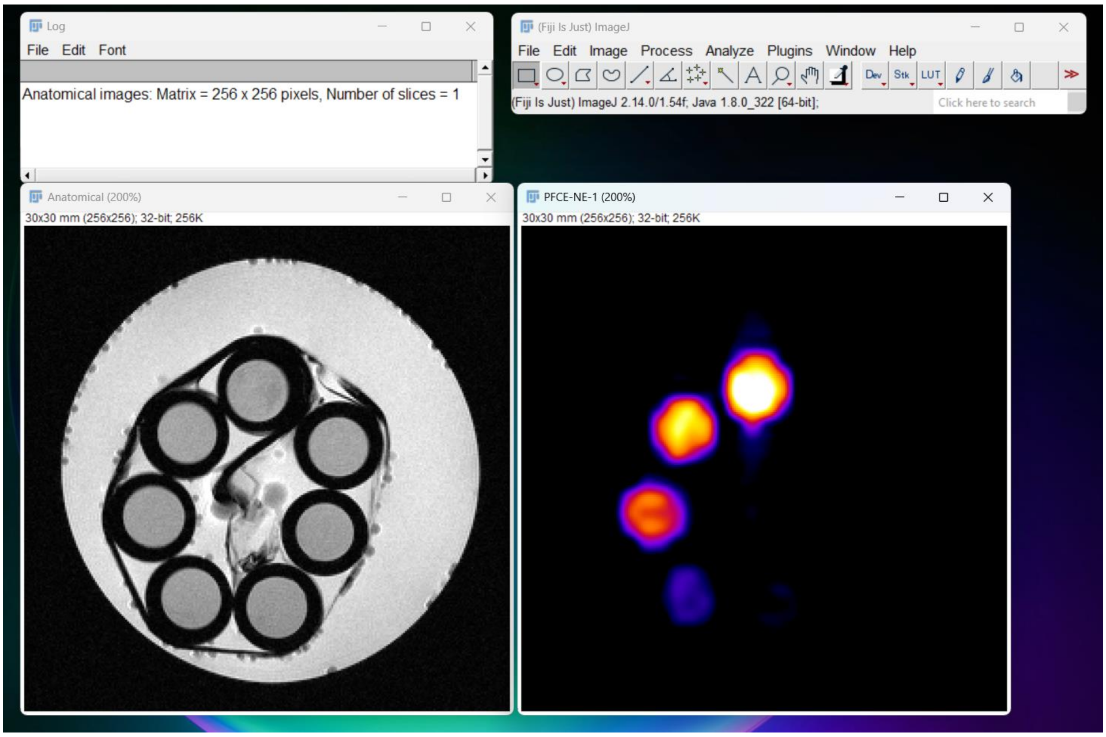

At this stage, the user can save, analyze the images, and create appropriate overlays.

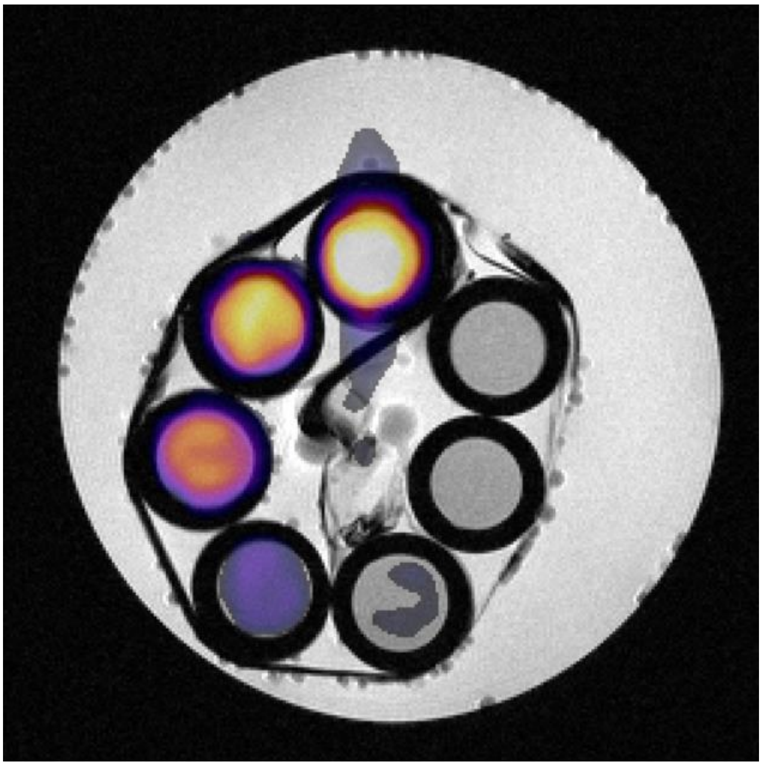

Supplement: Supplementary file 1 — Data S1. Methods: Preparation and characterization of the nanosystems; NMR characterization of the nanosystems; Shim protocol; Ex vivo experiments–Fluorescence microscopy; Ex vivo experiments–CSI of PLNs; In vivo CSI of PFCE‐NE–intravenous injection; 19F MRI vs. 1H CSI comparison. Figure S1. High‐resolution NMR spectra of DSPE‐PEG2000 in liposomes and micelles. Figure S2. High‐resolution NMR spectrum of Kolliphor® P188 in PFCE‐NE. Table S1. The measured SNR, the mean number of μmol of protons found in a single tumor slice (1 mm of thickness), and the % of moles of protons found in the tumor slice over the total injected proton moles are reported (n = 3). Figure S3. Hematoxylin and Eosin staining of PLNs. [file MRM-93-761-s001.zip › CSI_macros_guidelines.pdf]
